# Changes in dietary markers during the covid-19 pandemic in Brazil

**DOI:** 10.11606/s1518-8787.2023057004659

**Published:** 2023-09-04

**Authors:** Giovanna Calixto Andrade, Renata Bertazzi Levy, Maria Alvim Leite, Fernanda Rauber, Rafael Moreira Claro, Janine Giuberti Coutinho, Laís Amaral Mais

**Affiliations:** I Universidade de São Paulo Núcleo de Pesquisas Epidemiológicas em Nutrição e Saúde São Paulo SP Brazil Universidade de São Paulo. Núcleo de Pesquisas Epidemiológicas em Nutrição e Saúde. São Paulo, SP, Brazil; II Universidade de São Paulo Faculdade de Medicina Departamento de Medicina Preventiva São Paulo SP Brazil Universidade de São Paulo. Faculdade de Medicina. Departamento de Medicina Preventiva. São Paulo, SP, Brazil; III Universidade Federal de Minas Gerais Escola de Enfermagem Departamento de Nutrição Belo Horizonte MG Brasil Universidade Federal de Minas Gerais. Escola de Enfermagem. Departamento de Nutrição. Belo Horizonte, MG, Brasil; IV Instituto Brasileiro de Defesa do Consumidor São Paulo SP Brazil Instituto Brasileiro de Defesa do Consumidor. São Paulo, SP, Brazil

**Keywords:** Coronavirus, Eating. Feeding Behavior, Ultra-processed Foods

## Abstract

**OBJECTIVE:**

Evaluate changes in the Brazilian population’s diet and its determinants during the covid-19 pandemic.

**METHODS:**

We used diet data collected by the Datafolha Institute in 2019 (n = 1,384), 2020 (n =1,214), and 2021 (n = 1,459) from independent and representative samples of the adult population (aged 18 to 55 years) from all socioeconomic classes and geographic regions of Brazil. Food consumption was measured by checking the consumption of 22 sets of food on the day before the survey. The third cycle also included questions about changes in eating habits during the pandemic. We estimated the prevalence of consumption of the food sets in each cycle of the survey and used statistical tests for comparisons of proportions between the three cycles.

**RESULTS:**

Between 2019 and 2020, we observed a significant increase in the consumption of cereals, milk, packaged snacks or salty cookies, and industrialized sauces, as opposed to a decrease in the consumption of eggs. Between 2019 and 2021 and between 2020 and 2021, on the other hand, there was a significant decrease in the consumption of cereals, vegetables, fruits, and industrialized fruit juices and an increase in the consumption of soda, sweets, cookies, sausages, industrialized sauces, and ready meals. When asked about the main changes in the purchase and preparation of meals, 46.3% of the respondents reported consuming more food prepared at home during the pandemic. Regarding changes in eating habits, 48.6% of the respondents reported a change in their eating habits during the pandemic. The main reasons for such changes were greater concern with health (39.1%) and self-reported decreased family income (30.2%).

**CONCLUSIONS:**

The covid-19 pandemic had a negative impact on the diet of the population, and increased consumption of ultra-processed foods was reported for that period.

## INTRODUCTION

In December 2019, the first cases of infection by the Sars-CoV-2 coronavirus were reported in Asia. The virus spread rapidly around the world and reached Brazil in February 2020. The rapid geographical spread of the virus in a short period of time caused the World Health Organization (WHO) to declare it a pandemic on March 11, 2020^1^. Due to the large number of people infected and the lack of an effective vaccine or treatment against the disease at the time, health authorities around the world began to implement social distancing measures in order to reduce the spread of the virus^2^. This had a major impact on the lifestyle of the population, including changes in eating habits^3^.

In Brazil, restrictive measures were implemented starting in March 2020 and included suspension of all non-essential economic activities, school closures, and recommendations for people to stay in their homes^6^. In January 2021, after approval for emergency use, immunization was initiated through the administration of coronavirus vaccines. Due to the lack of coordination between the Ministry of Health, State and Municipal Health secretariats, vaccination advanced unevenly. Two years after the identification of the first case of covid-19 in the country, Brazil is still facing the pandemic and its economic and social consequences.

At the beginning of the pandemic, the Brazilian population was already in a situation of extreme vulnerability, with high unemployment rates, disruption of social policies and investments in health and research^7^. The pandemic worsened this scenario of vulnerability. Social isolation, job instability, and decreased income in some families impacted the access to food, increasing the risk of food insecurity^8^. Data from the “ *Inquérito nacional sobre insegurança alimentar no contexto da pandemia de covid-19 no Brasil* ” (National survey on food insecurity in the context of the covid-19 pandemic in Brazil) showed that the pandemic accelerated the prevalence of food insecurity in the country, which had been projected to increase since 2013^10^.

Changes in eating habits during the covid-19 pandemic have been reported by studies conducted in the country. A study carried out with data from the NutriNet Brazil cohort comparing the diet of more than 10,000 individuals in January 2020 (before the pandemic) and May 2020 (during the pandemic) pointed out changes in eating habits reported, in general, moderate, but statistically significant, increase in the consumption of healthy diet markers (including vegetables, fruits, and legumes) and stability in the consumption of ultra-processed foods. These changes, however, were not uniform in all population groups, and an increase in the consumption of ultra-processed food was reported in the Northeast and North regions, suggesting social inequalities in the response to the pandemic^3^. Another study conducted with 1,368 volunteers from all over the country aged 18 years or older, with data collected between August and September 2020, showed an increase in the frequency of fast food consumption, as opposed to a decrease in the consumption of fruits and vegetables^4^. Divergences between studies are probably a reflection of different methodologies applied, collection tools used, time period selected, and populations analyzed.

Despite indicating changes in eating habits, the studies conducted so far did not include representative samples of the population, did not assess more than one moment during the pandemic, and did not investigate the reasons for these changes. Thus, this study aims to investigate changes in dietary markers and the reasons for that during the covid-19 pandemic, in a representative sample of the Brazilian population.

## METHODS

This study evaluated markers of food consumption collected by the Datafolha Research Institute in 2019, 2020, and 2021, with independent samples representative of the adult population (men and women) aged 18 to 55 years, belonging to all socioeconomic classes and regions of Brazil.

### Sampling

Data collection occurred in three cycles, held respectively from October 7 to 11, 2019, June 4 to 13, 2020, and August 2 to 6, 2021. The first and third cycles followed an identical methodology, with personal interviews at points of high population flow. The intermediate cycle, due to restrictive measures resulting from the covid-19 pandemic, relied on data collection by telephone interview.

The sampling design of data collection at high flow points was based on information from the Census 2010/estimates 2016^11^, considering stratifications by Federal Units and size of municipalities. First, the municipalities were selected (n = 101) by simple random sampling and, subsequently, the points of high population flow in which the respondents were approached were also selected. In the flow point methodology, interviews are conducted on the street and in places with high traffic such as squares, intersections, avenues, and shopping streets, with a controlled approach through gender and age quotas. The points were drawn and distributed proportionally to the resident population, representing all the geographic regions of each surveyed area. Interviews were carried out with the aid of tablets, on weekdays and weekends. In all, 1,384 individuals were interviewed in 2019 and 1,459 in 2021.

For the 2nd cycle (conducted during a time of great restrictions attributed to the covid-19 pandemic), a database of cell phone numbers was initially generated through random digit dialing (RDD) from the list of prefixes of the Agência Nacional de Telecomunicações (the Brazilian Telecommunications Agency, Anatel) for mobile telephony. The database was validated, and non-existent numbers were discarded. In this methodology, all numbers have the same chance of being selected. Since the cell phone coverage is approximately 90% of the population, the collection by this mode has great potential to represent the Brazilian adult population. This cycle of the survey included a final sample of 1,214 individuals.

In the case of refusals, that is, individuals who did not want to participate in the study, these were randomly replaced by a subject with the same profile, considering the basic population quotas as a guide.

### Variables Description

In all survey cycles, food consumption was measured by means of two sets of questions about the consumption of food groups on the day before the survey.

The first set included 12 subgroups of fresh and minimally processed foods and their culinary preparations: (1) rice, noodles, polenta, couscous, or green corn; (2) potato, cassava, okra, or yam; (3) beans, peas, lentils, soybeans, or chickpeas; (4) beef, pork, chicken, or fish; (5) fried, boiled, or scrambled egg; (6) cabbage, broccoli, watercress, or other dark green leafy vegetable; (7) lettuce, chard, cabbage, or other light green leafy vegetables; (8) pumpkin, carrot, sweet potato, or okra; (9) tomato, cucumber, zucchini, or eggplant; (10) papaya, mango, yellow melon, or *pequi* ; (11) orange, banana, apple, or pineapple; and (12) milk. The second included ten subgroups of ultra-processed foods: (1) soda; (2) fruit juice in a box, carton, or can, or powdered soft drink; (3) chocolate drink or flavored yogurt; (4) crackers or salty snacks; (5) cookies; (6) ice cream, chocolate, gelatin, flan, or other industrialized dessert; (7) sausage, bologna, mortadella, ham, or other processed food; (8) buns, hot dogs, or hamburger buns; (9) margarine, mayonnaise, ketchup, or other processed sauces; and (10) instant noodles, packaged soup, frozen lasagna, or other ready-made frozen food. In both sets, the food subgroups were quoted by the interviewer and respondents were asked whether they had eaten them the previous day (yes or no). The food subgroups included in the questions were defined according to the NOVA^12^ classification groups. The sequence of the food subgroups within each set alternated among the interviews in order to minimize bias in the answers.

The third round of the survey included questions about changes in eating habits during the covid-19 pandemic, such as: “What was the main change in buying and preparing food during the pandemic? (I did not change my food buying and preparation; I started ordering more meals by delivery; I started eating more home cooked food; I started buying more frozen ready meals from the market)” and “What was the main reason for the changes in eating habits? (Did not change my diet; Decreased household budget; Increased household budget; More time available for cooking; Less time available for cooking; Increased concern for health; Social isolation/homework; Other).” Similarly, respondents were asked about changes in consumption of subgroups of fresh and minimally processed food and their culinary preparations and ultra-processed foods. They were instructed to report reduced, increased, or no change in consumption of: beef; pork; chicken; fish; fruits; vegetables and legumes; noodles; rice; beans; milk; egg; soft drinks; boxed, cartoned, or canned fruit juice or powdered soft drink; chocolate drink or flavored yogurt; packaged snack or saltine cracker/cooker; sweet, filled, or packaged cookie/cookie; ice cream, chocolate, gelatin, flan, or other processed dessert; sausage, bologna, bologna, ham, or other processed food; flatbread, hot dog, or hamburger buns; margarine, mayonnaise, ketchup, or other processed sauces; frozen ready meals (pizza, lasagna, etc.); and noodles or soups (e.g., pasta or soups); and instant noodles or soup.

In all cycles, socio-demographic information was collected, such as: gender (female; male), age group (18–24 years; 25–34 years; 35–44 years; 45–55 years), education (elementary; middle; higher), nature of the municipality (capital; interior; other municipalities of the metropolitan region), and region (Midwest; South; Southeast; North; Northeast).

### Data Analysis

To evaluate the population’s food consumption, the prevalence of individuals who reported consuming, on the day before the survey, each of the subgroups of fresh and minimally processed foods and their culinary preparations and the subgroups of ultra-processed food were calculated per survey cycle. The chi-square test was used for comparisons of proportions between the three survey cycles. The t-test was employed for comparisons of the number of subgroups consumed between cycles, adopting a p value ≤ 0.05 to identify statistically significant differences.

To assess perceptions of changes in food consumption and reasons motivating such changes, the major changes in food purchase and preparation during the covid-19 pandemic were estimated. For the most frequent reasons that motivated changes in food purchase, i.e., among individuals who reported as the main change “consuming more home-cooked food” and, among those who reported “ordering more meals for home delivery”, the proportion of changes in consumption of food subgroups (perceived increases, decreases, or changes in consumption) during this period was calculated.

Next, the main reasons driving changes in diet during the covid-19 pandemic were estimated. Changes in consumption (as per the procedure described) of food subgroups were analyzed among those who reported as reasons for changes in food consumption “increased health concern”, “decreased household budget”, and “more time available for cooking at home” (which represent the most frequent reasons for changes in food consumption during the covid-19 pandemic).

## RESULTS

The distribution of the population according to sociodemographic characteristics in the three cycles of the study is presented in Table 1 . In all cycles, most of the respondents were female, between 25 and 44 years old, had completed high school, lived in the capital cities or in other cities of the metropolitan regions in the Southeast region.

**Table 1:** Distribution of survey participants according to sociodemographic variables. Brazil, 2019, 2020, 2021.

Sociodemographic characteristics	Year of survey					

	2019		2020		2021	
		
	n	%	n	%	n	%
Sex						
Male	673	48.4	577	47.3	689	47.1
Female	711	51.6	637	52.7	770	52.9
Age group (years)						
18–24	278	19.7	216	22.4	299	19.5
25–34	379	27	343	27	401	27.3
35–44	377	27.9	353	26.7	405	28.1
45–55	350	25.4	302	23.9	354	25.1
Education						
Elementary school	339	25.4	391	35.7	332	24.3
Regular	719	51.7	468	48	721	49.8
Higher	326	22.9	355	16.4	406	25.9
Nature of the municipality						
Capital	384	24.3	415	26.8	381	24.8
Interior	760	57.9	593	58.7	818	58.1
Other municipalities of the metropolitan region	240	17.8	206	14.4	260	17.1
Region of residence						
Midwest	120	7.7	88	8.3	603	7.9
Northeast	359	26.2	336	27.2	221	26
North	118	7.9	102	8.9	392	8.1
Southeast	586	43.5	518	41.6	123	43.1
South	201	14.7	170	14	120	14.9
Total	1,384	100	1,214	100	1,459	100

Table 2 displays food consumption in 2019, 2020, and 2021. In the three cycles, the most consumed subgroups of fresh and minimally processed foods (and their culinary preparations) were cereals (rice, pasta, polenta, corn, or couscous) and legumes (beans, peas, lentils, soybeans, or chickpeas). Among the ultra-processed foods, the consumption of margarine and industrialized sauces (mayonnaise, ketchup, or other industrialized sauces), soft drinks, and sausages (sausage, bologna, mortadella, ham, or other processed food) stands out. Comparing food consumption in the three cycles of the survey, between 2019 and 2020 there is a significant increase in the consumption of cereals, milk, packaged snacks or crackers and margarine and industrialized sauces, as opposed to a decrease in the consumption of eggs. Between 2019 and 2021 and between 2020 and 2021, on the other hand, there was a significant decrease in the consumption of cereals, vegetables, fruits, and industrialized fruit juices (boxed, carton, or canned fruit juice or powdered soft drink) and an increase in the consumption of soft drinks, sweet, filled, or packaged cookies, sausages, industrialized margarine and sauces, and ready meals (instant noodles, packaged soup, frozen lasagna, or other ready meal purchased frozen).

**Table 2:** Prevalence of food consumption on the day before the survey. Brazil, 2019, 2020 and 2021.

Food groups	Year of survey					

	2019		2020		2021	
		
	%	SE	%	SE	%	SE
Fresh, minimally processed foods and culinary preparations						
Rice, noodles, polenta, corn, or couscous	83.8	1	91.3^a^	0.9	87.7^a,b^	0.9
Potato, cassava, okra, or yam	42.4	1.3	39.1	1.6	43	1.3
Beans, peas, lentils, soybeans, or chickpeas	75.8	1.2	75.9	1.4	74.5	1.2
Pork, beef, chicken, or fish	86	0.9	86.5	1.1	86.7	0.9
Fried, boiled, or scrambled egg	45.4	1.3	41.2^a^	1.6	44.1	1.3
Vegetables	80.6	1.1	80.7	1.3	75.8^a,b^	1.2
Fruits	67.8	1.3	65	1.6	61.3^a^	1.3
Milk	54.2	1.4	59.0^a^	1.6	56	1.3
Ultra-processed foods						
Soda	33.2	1.3	32.7	1.5	42.4^a,b^	1.3
Fruit juice in a box, carton or can, or powdered soft drink	29.7	1.2	29.3	1.5	26.3^a^	1.2
Chocolate drink or flavored yogurt	21.7	1.1	22.1	1.3	21.9	1.1
Crackers or salty snacks	30.1	1.2	35.0^a^	1.6	32	1.2
Cookies	33	1.3	31.3	1.5	36.7^a,b^	1.3
Ice cream, chocolate, gelatin, flan, or other industrialized dessert	24.4	1.2	21.8	1.3	22.1	1.1
Sausage, chorizo, mortadella, ham, or other processed food	31.1	1.3	34.4	1.5	37.5^a^	1.3
Loaf of bread, hot dog, or hamburger bun	29.3	1.2	29.1	1.5	32.1	1.2
Margarine, mayonnaise, ketchup, or other industrialized sauces	50.2	1.4	54.4^a^	1.6	58.6^a,b^	1.3
Instant noodles, packaged soup, frozen lasagna, or other ready-made frozen food	9.7	0.8	10.9	1	13.9^a,b^	0.9

Source: data from the Datafolha survey, carried out with representative samples of the Brazilian adult population. For more information, see methods.

SE: standard error.

^a^ p ≤ 0.05 (reference year: 2019).

^b^ p ≤ 0.05 (reference year: 2020).

**Figure 1:**
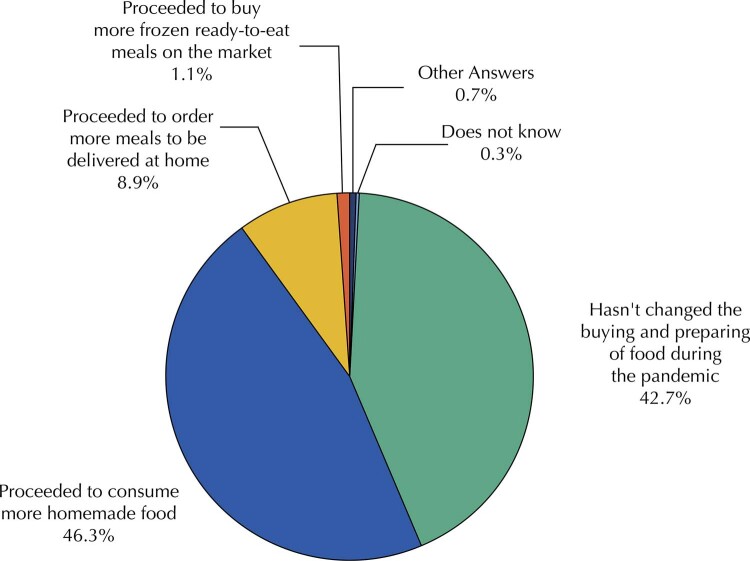
Perceived changes in food purchasing and preparation during the covid-19 pandemic. Brazil, 2021.

Figure 1 presents the distribution of individuals according to the main changes in food purchase and preparation during the covid-19 pandemic. When asked, 46.3% of respondents reported that they consumed more food prepared at home, 43.0% said there was no change in food purchasing or preparation patterns, and 9% reported an increase in ordering meals for home delivery.

Table 3 presents the changes in diet during the covid-19 pandemic among individuals who reported “consuming more home-cooked food” and “ordering more meals for home delivery”. Among those who consumed more home-cooked meals during the pandemic, a higher proportion of participants reported increased consumption of egg, chicken, fruits, vegetables, and legumes and individuals who reported decreased consumption of beef. Additionally, a higher proportion of individuals reported decreased consumption of all subgroups of ultra-processed foods, compared to the proportion of participants who reported increased consumption of these foods. Among those who reported ordering more meals for home delivery during the pandemic, the proportion of participants who reported increased consumption of pork, egg, soda, and frozen ready meals and individuals who reported a decrease in beef consumption stands out.

**Table 3:** Perception of changes in food consumption (increase, decrease or unchanged) during the covid-19 pandemic among individuals who elected, as the main change in food purchase and preparation, “consume more home-made food” and “order more meals for home delivery”. Brazil, 2021.

Variables	Individuals who reported consuming more home-delivered food during the covid-19 pandemic (n = 674)			Individuals who reported ordering more meals for home delivery during the covid-19 pandemic (n = 133)		
	
	Increased	Decreased	Did not change	Increased	Decreased	Did not change
					
	(%)	(%)	(%)	(%)	(%)	(%)
Fresh, minimally processed foods and culinary preparations						
Beef	22.3	47.8	29.8	20.1	40.8	39.1
Pork	19.8	34.6	45.6	20.2	33.4	46.4
Chicken	53.8	13.4	32.9	53.2	13	33.8
Fish	26.2	31.3	42.6	21.8	26.6	51.6
Fruits	49.2	19.8	31.0	37.4	21.2	41.4
Vegetables and legumes	47.2	19.0	33.9	30.5	22.5	46.9
Noodles	29.1	28.6	42.3	34.0	18.8	47.3
Rice	37.7	15.4	46.9	31.4	16.8	51.7
Bean	37.9	14.9	47.2	29.6	19.8	50.6
Milk	29.2	29.1	41.6	29.3	20.1	50.7
Egg	54.4	11.3	34.3	49.9	12.3	37.8
Ultra-processed foods						
Soda	17.5	47.5	35	44	24.6	31.3
Fruit juice in a box, carton or can, or powdered soft drink	16.4	39.1	44.5	29.5	22.3	48.2
Chocolate drink or flavored yogurt	10.2	43.3	46.6	27.2	20.7	52.2
Crackers or salty snacks	15	44.7	40.3	29.3	27.3	43.4
Cookies	16.3	41.3	42.4	28.3	28	43.7
Ice cream, chocolate, gelatin, flan, or other industrialized dessert	11.9	46.6	41.5	28.3	22.7	49
Sausage, chorizo, mortadella, ham, or other processed food	21.4	38.4	40.1	35.2	22.7	42.1
Loaf of bread, hot dog, or hamburger bun	20.1	40.2	39.7	39.1	20	40.8
Margarine, mayonnaise, ketchup, or other industrialized sauces	16	37	47	36.4	18.7	44.9
Frozen ready to eat meals (pizza, lasagna, etc.)	8.6	46.2	45.2	44.4	21.7	33.9
Instant noodles or soup	19.9	34.1	46	28.7	28.9	42.4

Source: data from the Datafolha survey carried out with representative samples of the Brazilian adult population. For more information, see methods.

**Figure 2:**
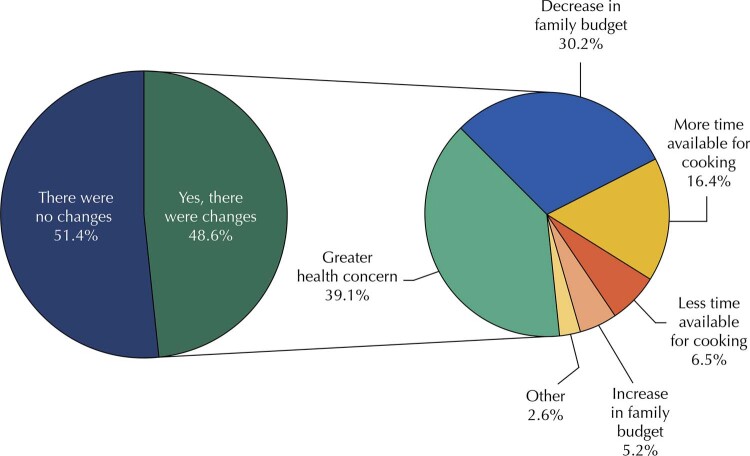
Perceived changes in eating habits and the main reasons why these changes happened during covid-19 pandemic, Brazil, 2021.

Figure 2 presents the distribution of individuals according to the perception of changes in eating habits whose main reasons for changes occurred during the covid-19 pandemic. When asked, 51.4% of respondents reported they had not changed their eating habits during the pandemic. Moreover, among those who reported changes (48.6%), the most frequent reason was greater concern for health (39.1%), followed by decreased household income (30.2%) and more time available for cooking (16.4%).

Table 4 presents the perceptions of changes in consumption of food groups and subgroups during the covid-19 pandemic among individuals who chose as the main reason for such changes “increased health concern”, “decreased household budget”, and “more time available for cooking at home”. Among those who reported more concern for health, there was an increase in the consumption of chicken, fruits, vegetables, and egg, and a decrease in the consumption of beef and all subgroups of ultra-processed foods. Among those who reported a decrease in their household budget, there is an increase in the consumption of chicken and egg and a decrease in the consumption of beef, pork, fish, fruit, milk and all the subgroups of ultra-processed foods. Among those who reported more time available for cooking at home, there was an increase in the consumption of chicken, fruits, vegetables, rice, beans, and egg. Additionally, among these individuals, most reported no change in the consumption of ultra-processed food groups.

**Table 4:** Perception of changes in food consumption (increase, decrease or changes) during the covid-19 pandemic among individuals who elected as the main reason for such changes “greater concern with health”, “decrease in family budget” and “more time available for cooking at home”. Brazil, 2021.

Variables	Individuals who reported increased health concerns as the main cause of dietary change during the covid-19 pandemic (n = 288)			Individuals who reported decreased household budget as the main cause of dietary change during the covid-19 pandemic (n = 207)			Individuals who reported having more time to cook at home as the main cause of dietary change during the covid-19 pandemic (n = 123)		
		
	Increased	Decreased	Did not change	Increased	Decreased	Did not change	Increased	Decreased	Did not change
								
	(%)	(%)	(%)	(%)	(%)	(%)	(%)	(%)	(%)
Fresh, minimally processed foods and culinary preparations									
Beef	23.4	48.7	27.8	7.1	76	16.8	33.5	28.1	38.4
Pork	16.6	38.6	44.8	17.9	50.5	31.6	23.7	22.7	53.6
Chicken	58.7	12.7	28.6	49.2	27.5	23.3	62.6	6.4	31
Fish	32.4	25.8	41.9	14.1	49.3	36.6	34.8	24	41.3
Fruits	63.6	13.6	22.8	31.8	42	26.2	64	10.5	25.5
Vegetables and legumes	63.6	12.8	23.6	34.5	34	31.4	68.1	10.6	21.2
Noodles	27.4	33.9	38.7	30.8	32.2	37	26.9	23.1	50
Rice	33.1	27.4	39.5	33.4	20.9	45.7	49.3	10.9	39.8
Bean	39.3	17	43.7	31.3	26.9	41.8	44.3	8.5	47.3
Milk	30.7	26.2	43.1	25.1	40.3	34.6	37.2	17.3	45.5
Egg	53.5	15	31.4	59.2	13.3	27.5	63.5	7.2	29.3
Ultra-processed foods									
Soda	19.4	50.7	29.9	16.2	58.2	25.6	25.6	33.2	41.2
Fruit juice in a box, carton or can, or powdered soft drink	14.9	44	41.2	18.5	47.9	33.6	23.7	26.8	49.5
Chocolate drink or flavored yogurt	11.7	43.6	44.8	7.9	55.8	36.4	20.3	31	48.7
Crackers or salty snacks	11.4	52.9	35.7	19.4	54.7	25.9	25.2	36.1	38.7
Cookies	16.5	45.1	38.4	18.6	55.5	25.8	25.1	32.3	42.6
Ice cream, chocolate, gelatin, flan, or other industrialized dessert	11.2	47.9	40.9	7.8	61	31.1	31.5	28.7	39.8
Sausage, chorizo, mortadella, ham, or other processed food	17.3	44	38.7	28.7	43.9	27.4	28.5	24.5	47
Loaf of bread, hot dog, or hamburger bun	21.4	39.2	39.5	19.1	48.7	32.2	27.9	24.4	47.7
Margarine, mayonnaise, ketchup, or other industrialized sauces	16.9	43.2	39.9	15.8	44.6	39.5	26.3	28.1	45.6
Frozen ready to eat meals (pizza, lasagna, etc.)	7.7	47.1	45.2	10.1	60.1	29.9	23.8	35.1	41.1
Instant noodles or soup	16.8	42.6	40.6	21.2	39.2	39.6	14.6	30.9	54.5

Source: data from the Datafolha survey carried out with representative samples of the Brazilian adult population. For more information, see methods.

## DISCUSSION

The results of this study suggest negative changes in the diet of the Brazilian population during the covid-19 pandemic. However, different changes were observed at different stages of the pandemic.

In the first cycle of the survey, conducted before the pandemic (in 2019), more than 75% of participants reported consumption, on the day before the interview, of at least one type of meat, cereal, vegetable, or legume. Ultra-processed food groups, although reported by a significant portion of the population, appeared in a lower proportion of consumption. These results are in line with national surveys which indicate that, despite a growing increase in the consumption of ultra-processed foods, the basis of the Brazilian diet is still composed of fresh and minimally processed foods, especially the consumption of rice, beans, and meat^13^.

The second cycle of the survey indicated that, during the pandemic, there were changes in food consumption by Brazilians. In 2020, about three months after the beginning of the confinement period, we observed an increase in the proportion of individuals who reported consumption of cereals and milk on the day before the survey. An increase was also observed in the consumption of packaged snacks or salted biscuits/ crackers and industrialized margarine and sauces, suggesting increased consumption of both healthy and unhealthy markers of diet.

These results are comparable to other surveys conducted in the country in the first months after the implementation of restrictive measures. Among participants in the NutriNet Brazil cohort, an increase in consumption of healthy dietary markers was generally observed, but among lower socioeconomic strata the opposite trend occurred, as an increase in consumption of unhealthy dietary markers was reported^3^. Furthermore, an observational study in a convenience sample reported increased consumption of fast food and ready to eat meals versus decreased consumption of fruit^4^. Comparisons with the literature, however, are limited because studies used different methods for sample selection and data collection.

The comparison between the first cycles (2019 and 2020) and the third cycle of the survey (2021) shows an increase in the consumption of ultra-processed foods, especially sodas, crackers, sausages, margarine and industrialized sauces, and ready to eat meals, as opposed to a decrease in the consumption of fresh and minimally processed foods and their culinary preparations, especially fruits and vegetables. Due to the short period between surveys, changes were small but statistically significant. These results are worrisome, since the consumption of ultra-processed foods has been associated with a decline in the nutritional profile of the diet^14^. Additionally, studies show an association between consumption of ultra-processed foods and negative health outcomes, such as obesity, metabolic syndrome, diabetes, and different types of cancer^15^. Obesity and other chronic non-communicable diseases (NCDs), in turn, are risk factors for worsening symptoms in people infected with covid-19^19 , 20^.

Changes in diet during the covid-19 pandemic can be attributed to different reasons. As noted in the results on perceptions of dietary changes during confinement, most respondents who reported “increased health concerns” stated that they increased their consumption of fruits and vegetables and decreased their consumption of ultra-processed foods during that period. Similar changes were found among individuals who reported “consuming more prepared foods at home” as the main change in food purchase and preparation during the pandemic. Among respondents who reported “ordering more meals for home delivery,” on the other hand, mostly said they had increased consumption of ultra-processed foods such as sodas and frozen ready to eat meals.

Other factors not evaluated in this study may also explain the increased consumption of ultra-processed foods. The advertising of these products, for example, is one of the factors that may have boosted their consumption during the covid-19 pandemic. Studies show that the ultra-processed food industry took advantage of the global context to promote their products as “advantageous” during the pandemic, since they are less perishable and thus could contribute to social distancing, appealing to consumers’ social responsibility and linking their image to health^21 , 22^.

The increased consumption of ultra-processed foods may also be linked to the negative impact of social distancing on the population’s mental health. Studies conducted in Brazil indicate a high occurrence of psychological symptoms, such as depression, anxiety, and stress during the pandemic^23 , 24^. Mental health disorders, in turn, have been associated with unhealthy diets^25^, so psychological symptoms may partly explain the increased consumption of these foods during the period of social withdrawal.

Other factors, such as increased food prices^29^, increased rates of unemployed population^30^, and reduced emergency wage^10^ during the pandemic may also explain changes in food consumption. In this study, individuals who reported “decreased household budget” as the main cause of dietary change during the covid-19 pandemic mostly reported reduced consumption of fresh and minimally processed foods such as beef and pork, fish, fruit, and milk, in addition to consumption of all ultra-processed food groups. This result suggests difficulty of access to food by a significant portion of the population. Such data goes against the results of the “ *Inquérito nacional sobre insegurança alimentar no contexto da pandemia da covid-19 no Brasil* ” that show that the pandemic accelerated the prevalence of food insecurity in the country, which had been projected to grow since 2013. In Brazil, between 2018 and 2020, mild food insecurity increased from 20.7% to 34.7%; moderate food insecurity increased from 10.1% to 11.5%; and severe food insecurity increased from 5.8% to 9%^10^.

Among the limitations of this study, we highlight the measurement of the population’s food consumption that was carried out through simplified questionnaires in specific periods, which disregarded the annual seasonality of food production. Additionally, due to circulation restrictions resulting from the pandemic, the method for collecting the information was different in the second cycle of the survey, although the food consumption questionnaires were the same in the three cycles. It is also worth noting that the results were partly based on perceptions of changes in food consumption, which do not necessarily reflect actual changes. Among the positive points of the study, we can mention its representative sample of the Brazilian population. In addition, this is the first published study to investigate changes in diet at two distinct points in the covid-19 pandemic and to investigate the reasons for such changes.

Results from this study will help to understand the effects of the covid-19 pandemic on population diet, and to understand the reasons for such changes. Increased consumption of ultra-processed foods is already a phenomenon reported in Brazil by national studies conducted periodically, however, this study, as well as others^3 , 4^, suggests that the pandemic accelerated this phenomenon, negatively impacting the population’s diet. However, more research is needed to assess the magnitude and long-term effects of the covid-19 pandemic on Brazilians’ diets.
