# Supplementary material for: Changes in dietary markers during the covid-19 pandemic in Brazil
Source: Rev Saude Publica. 2023 Sep 4;57:54. doi: 10.11606/s1518-8787.2023057004659 (PMC10519684; doi:10.11606/s1518-8787.2023057004659)
Supplement: Translation

# Translation: Mudanças nos marcadores da alimentação durante a pandemia de covid-19 no Brasil

**Authors:** Giovanna Calixto Andrade, Renata Bertazzi Levy, Maria Alvim Leite, Fernanda Rauber, Rafael Moreira Claro, Janine Giuberti Coutinho, Laís Amaral Mais

**Affiliations:** I Brazil Universidade de São Paulo. Núcleo de Pesquisas Epidemiológicas em Nutrição e Saúde. São Paulo, SP, Brazil; II Brazil Universidade de São Paulo. Faculdade de Medicina. Departamento de Medicina Preventiva. São Paulo, SP, Brazil; III Brasil Universidade Federal de Minas Gerais. Escola de Enfermagem. Departamento de Nutrição. Belo Horizonte, MG, Brasil; IV Brazil Instituto Brasileiro de Defesa do Consumidor. São Paulo, SP, Brazil

**Keywords:** Coronavírus, Ingestão de Alimentos, Comportamento Alimentar, Alimento Ultraprocessado

## Abstract

**OBJETIVO:**

Analisar mudanças na alimentação da população brasileira e seus determinantes durante a pandemia de covid-19.

**MÉTODOS:**

Foram utilizados dados de alimentação coletados pelo instituto Datafolha, em 2019 (n = 1.384), 2020 (n = 1.214) e 2021 (n = 1.459), de amostras independentes e representativas da população adulta (entre 18 e 55 anos) de todas as classes socioeconômicas e regiões geográficas do Brasil. O consumo alimentar foi aferido por meio da verificação do consumo de 22 conjuntos de alimentos no dia anterior à pesquisa. No terceiro ciclo, também foram incluídas questões sobre mudanças nos hábitos alimentares durante a pandemia. Estimou-se a prevalência de consumo dos conjuntos de alimentos em cada ciclo da pesquisa e foram utilizados testes estatísticos para comparações de proporções entre os três ciclos.

**RESULTADOS:**

Observou-se, entre 2019 e 2020, aumento significativo no consumo de cereais, leite, salgadinhos de pacote ou biscoitos salgados e molhos industrializados, em contraponto à diminuição do consumo de ovos. Entre 2019 e 2021 e entre 2020 e 2021, por outro lado, houve diminuição significativa no consumo de cereais, hortaliças, frutas e sucos de fruta industrializados e aumento no consumo de refrigerante, biscoito doce, recheado ou bolinho de pacote, embutidos, molhos industrializados e refeições prontas. Quando questionados sobre as principais mudanças na compra e preparo das refeições, 46,3% dos entrevistados relataram consumir mais alimentos preparados em casa durante a pandemia. Em relação a mudanças nos hábitos alimentares, 48,6% dos entrevistados relataram alteração na alimentação durante a pandemia. Os principais motivos para tais mudanças foram maior preocupação com a saúde (39,1%) e autorrelato de diminuição da renda familiar (30,2%).

**CONCLUSÕES:**

A pandemia de covid-19 teve impacto negativo na alimentação da população, e foi reportado aumento no consumo de alimentos ultraprocessados durante esse período.

## INTRODUÇÃO

Em dezembro de 2019, foram reportados, na Ásia, os primeiros casos de infecção causada pelo coronavírus Sars-CoV-2. O vírus espalhou-se rapidamente ao redor do mundo e chegou ao Brasil em fevereiro de 2020. A rápida disseminação geográfica do vírus em um curto período fez com que a Organização Mundial da Saúde (OMS) declarasse o quadro como pandêmico no dia 11 de março de 2020^1^. Pelo grande número de infectados e pela falta de vacina ou tratamento eficaz contra a doença na época, as autoridades de saúde ao redor do mundo começaram a implementar medidas de distanciamento social, com o intuito de reduzir a propagação do vírus^2^, o que causou grande impacto no estilo de vida da população, incluindo mudanças nos hábitos alimentares^3^.

No Brasil, medidas restritivas foram implementadas a partir de março de 2020 e incluíram suspensão de todas as atividades econômicas não essenciais, fechamento de escolas e recomendações para que as pessoas ficassem em seus domicílios^6^. Em janeiro de 2021, após aprovação para uso emergencial, foi iniciada a imunização por meio da administração de vacinas contra o coronavírus. Devido à desarticulação do Ministério da Saúde com as secretarias estaduais e municipais de saúde, a vacinação avança de maneira não equânime. Dois anos depois da identificação do primeiro caso de covid-19 no país, o Brasil ainda enfrenta a pandemia e suas consequências econômicas e sociais.

No início da pandemia, a população brasileira já se encontrava em situação de extrema vulnerabilidade, com altas taxas de desemprego, desestruturação nas políticas sociais e em investimentos em saúde e pesquisas^7^. O quadro pandêmico agravou tal cenário de vulnerabilidade. O isolamento social, a instabilidade no trabalho e a diminuição da renda em algumas famílias impactaram o acesso ao alimento, aumentando o risco de insegurança alimentar^8^. Dados do “Inquérito nacional sobre insegurança alimentar no contexto da pandemia de covid-19 no Brasil” mostraram que a pandemia acelerou a prevalência de insegurança alimentar no país, que apresentava projeções de crescimento desde 2013^10^.

Mudanças nos hábitos alimentares durante a pandemia de covid-19 têm sido relatadas por estudos conduzidos no país. Estudo realizado com dados da coorte NutriNet Brasil comparando a alimentação de mais de 10 mil indivíduos em janeiro de 2020 (antes da pandemia) e maio de 2020 (durante a pandemia) apontou mudanças nos hábitos alimentares reportou, de maneira geral, aumento moderado, mas estatisticamente significativo, no consumo de marcadores saudáveis da alimentação (incluindo hortaliças, frutas e leguminosas) e estabilidade no consumo de alimentos ultraprocessados. Tais mudanças, entretanto, não foram uniformes em todos os grupos populacionais, e foi relatado, nas regiões Nordeste e Norte, aumento no consumo de alimentos ultraprocessados, sugerindo desigualdades sociais na resposta à pandemia^3^. Outro estudo realizado com 1.368 voluntários de todo o país com 18 anos ou mais, com dados coletados entre agosto e setembro de 2020, apontou aumento na frequência de consumo de *fast food* , em contraponto à diminuição do consumo de frutas e hortaliças^4^. Divergências entre os estudos são provavelmente reflexo de diferentes metodologias aplicadas, instrumentos de coleta utilizados, período de tempo selecionado e populações analisadas.

Apesar de indicarem mudanças nos hábitos alimentares, os estudos realizados até o momento não incluíram amostras representativas da população, não avaliaram mais de um momento durante a pandemia e não investigaram os motivos dessas mudanças. Assim, este estudo tem como objetivo investigar mudanças de marcadores da alimentação e suas razões durante a pandemia de covid-19, em uma amostra representativa da população brasileira.

## MÉTODOS

O presente estudo avaliou marcadores do consumo alimentar coletados pelo instituto de pesquisa Datafolha em 2019, 2020 e 2021, com amostras independentes representativas da população adulta (homens e mulheres) com idade entre 18 e 55 anos, pertencentes a todas as classes socioeconômicas e regiões do Brasil.

### Amostragem

A coleta de dados ocorreu em três ciclos, realizados respectivamente de 7 a 11 de outubro de 2019, de 4 a 13 de junho de 2020 e de 2 a 6 de agosto de 2021. O primeiro e o terceiro ciclos seguiram metodologia idêntica, com entrevistas pessoais em pontos de alto fluxo populacional. Já o ciclo intermediário, devido a medidas restritivas decorrentes da pandemia de covid-19, contou com a coleta de dados por entrevista telefônica.

O desenho amostral das coletas de dados em pontos de alto fluxo foi elaborado com base em informações do Censo 2010/estimativa 2016^11^, considerando estratificações por Unidades Federativas e porte dos municípios. Primeiramente, por amostragem aleatória simples, foram selecionados os municípios (n = 101) e, posteriormente, os pontos de alto fluxo populacional nos quais os entrevistados foram abordados. Na metodologia de ponto de fluxo, as entrevistas são realizadas na rua e em locais de alta movimentação, tais como praças, cruzamentos, avenidas e ruas de comércio, com abordagem controlada por meio de cotas de sexo e idade. Os pontos foram sorteados e distribuídos proporcionalmente à população residente de forma representativa de todas as regiões geográficas de cada área pesquisada. As entrevistas foram realizadas com auxílio de tablets, em dias úteis e finais de semana. Ao todo, foram entrevistados 1.384 indivíduos em 2019 e 1.459 em 2021.

Já para o 2º ciclo (realizado durante um momento com grandes restrições atribuídas à pandemia da covid-19), um banco de dados de números de telefones celulares foi inicialmente gerado por meio de discagem aleatória de dígitos ( *random digit dialing* – RDD) a partir da relação de prefixos da Agência Nacional de Telecomunicações (Anatel) de telefonia celular. Esse banco de dados foi validado e números inexistentes foram descartados. Nessa metodologia, todos os números apresentam a mesma chance de serem selecionados. Uma vez que a cobertura da telefonia celular é de aproximadamente 90% da população, a coleta por esse modal tem grande potencial para representar a população adulta brasileira. Esse ciclo da pesquisa incluiu uma amostra final de 1.214 indivíduos.

No caso de recusas, isto é, de indivíduos que não quiseram participar do estudo, esses foram substituídos de forma aleatória por um correspondente de igual perfil, considerando as cotas básicas da população como guia.

### Descrição das Variáveis

Em todos os ciclos da pesquisa, o consumo alimentar foi aferido por meio de dois conjuntos de perguntas sobre o consumo de grupos de alimentos no dia anterior à pesquisa.

O primeiro conjunto incluiu 12 subgrupos de alimentos *in natura* e minimamente processados e suas preparações culinárias: 1) arroz, macarrão, polenta, cuscuz ou milho-verde; 2) batata, mandioca, cará ou inhame; 3) feijão, ervilha, lentilha, soja ou grão-de-bico; 4) carne de boi, porco, frango ou peixe; 5) ovo frito, cozido ou mexido; 6) couve, brócolis, agrião ou outra verdura de folha verde-escura; 7) alface, acelga, repolho ou outra verdura de folha verde-clara; 8) abóbora, cenoura, batata-doce ou quiabo; 9) tomate, pepino, abobrinha ou berinjela; 10) mamão, manga, melão amarelo ou pequi; 11) laranja, banana, maçã ou abacaxi; e 12) leite. Já o segundo incluiu dez subgrupos de alimentos ultraprocessados: 1) refrigerante; 2) suco de fruta em caixa, caixinha ou lata ou refresco em pó; 3) bebida achocolatada ou iogurte com sabor; 4) salgadinho de pacote ou biscoito/bolacha salgada; 5) biscoito/bolacha doce, recheada ou bolinho de pacote; 6) sorvete, chocolate, gelatina, flan ou outra sobremesa industrializada; 7) salsicha, linguiça, mortadela, presunto ou outro alimento embutido; 8) pão de forma, de cachorro-quente ou de hambúrguer; 9) margarina, maionese, ketchup ou outros molhos industrializados; e 10) macarrão instantâneo, sopa de pacote, lasanha congelada ou outro prato pronto comprado congelado. Em ambos os conjuntos, os subgrupos de alimentos eram citados pelo entrevistador e os entrevistados respondiam quanto ao consumo no dia anterior (sim ou não). Os subgrupos de alimentos incluídos nas questões foram definidos respeitando os grupos da classificação NOVA^12^. A sequência dos subgrupos de alimentos dentro de cada conjunto se alternava entre as entrevistas de forma a minimizar o vício nas respostas.

O terceiro ciclo da pesquisa incluiu questões sobre mudanças nos hábitos alimentares durante a pandemia de covid-19, como: “Qual foi a principal mudança na compra e no preparo de alimentos durante a pandemia? (Não mudei a compra e o preparo de alimentos; Passei a pedir mais refeições por delivery; Passei a consumir mais comida feita em casa; Passei a comprar mais refeições prontas congeladas no mercado)” e “Qual foi o principal motivo das mudanças na alimentação? (Não mudei minha alimentação; Diminuição do orçamento familiar; Aumento do orçamento familiar; Mais tempo disponível para cozinhar; Menos tempo disponível para cozinhar; Maior preocupação com a saúde; Isolamento social/trabalho de casa; Outro)”. De forma análoga, os entrevistados foram questionados sobre mudanças no consumo de subgrupos de alimentos *in natura* e minimamente processados e suas preparações culinárias e alimentos ultraprocessados, sendo orientados a relatar redução, aumento ou não alteração no consumo de: carne de boi; carne de porco; frango; peixe; frutas; legumes e verduras; macarrão; arroz; feijão; leite; ovo; refrigerante; suco de fruta em caixa, caixinha ou lata ou refresco em pó; bebida achocolatada ou iogurte com sabor; salgadinho de pacote ou biscoito/bolacha salgada; biscoito/bolacha doce, recheada ou bolinho de pacote; sorvete, chocolate, gelatina, flan ou outra sobremesa industrializada; salsicha, linguiça, mortadela, presunto ou outro alimento embutido; pão de forma, de cachorro-quente ou de hambúrguer; margarina, maionese, ketchup ou outros molhos industrializados; refeições prontas congeladas (pizza, lasanha etc.); e macarrão ou sopa instantâneos.

Em todos os ciclos, foram coletadas informações sociodemográficas, como: sexo (feminino; masculino), faixa etária (18–24 anos; 25–34 anos; 35–44 anos; 45–55 anos), escolaridade (fundamental; médio; superior), natureza do município (capital; interior; outros municípios da região metropolitana) e região (Centro-Oeste; Sul; Sudeste; Norte; Nordeste).

### Análises de Dados

Para avaliar o consumo alimentar da população, foram calculadas as prevalências de indivíduos que relataram consumir, no dia anterior à pesquisa, cada um dos subgrupos de alimentos *in natura* e minimamente processados e suas preparações culinárias e os subgrupos de alimentos ultraprocessados por ciclo de pesquisa. O teste qui-quadrado foi utilizado para comparações de proporções entre os três ciclos da pesquisa. O teste t foi empregado para comparação do número de subgrupos consumidos entre os ciclos, adotando-se o valor de p ≤ 0,05 para identificar diferenças estatisticamente significativas.

Para avaliar percepções de mudanças no consumo de alimentos e razões que motivaram tais alterações, foram estimadas as principais mudanças na compra e no preparo de alimentos durante a pandemia da covid-19. Para as razões mais frequentes que motivaram alterações na compra de alimentos, ou seja, entre os indivíduos que relataram como principal mudança “consumir mais comida feita em casa” e, entre aqueles que relataram “pedir mais refeições para entrega em casa”, calculou-se a proporção das alterações no consumo de subgrupos de alimentos (percepção de aumento, redução ou inalterações no consumo) durante esse período.

Posteriormente, foram estimados os principais motivos que impulsionaram mudanças na alimentação durante a pandemia de covid-19. Alterações no consumo (conforme procedimento descrito) de subgrupos de alimentos foram analisadas entre aqueles que referiram como razões para mudança no consumo alimentar “maior preocupação com saúde”, “diminuição do orçamento familiar” e “mais tempo disponível para cozinhar em casa” (que representam as razões mais frequentes para mudanças no consumo alimentar durante a pandemia de covid-19).

## RESULTADOS

A distribuição da população segundo características sociodemográficas nos três ciclos da pesquisa é apresentada na Tabela 1 . Em todos os ciclos, a maioria dos entrevistados era do sexo feminino, tinha entre 25 e 44 anos, com ensino médio completo, residia nas capitais, ou em outros municípios de regiões metropolitanas, na região Sudeste.

**Tabela 1:** Distribuição dos participantes da pesquisa segundo variáveis sociodemográficas. Brasil, 2019, 2020, 2021.

Características sociodemográficas	Ano da pesquisa					

	2019		2020		2021	
		
	n	%	n	%	n	%
Sexo						
Masculino	673	48,4	577	47,3	689	47,1
Feminino	711	51,6	637	52,7	770	52,9
Faixa etária (anos)						
18–24	278	19,7	216	22,4	299	19,5
25–34	379	27	343	27	401	27,3
35–44	377	27,9	353	26,7	405	28,1
45–55	350	25,4	302	23,9	354	25,1
Escolaridade						
Fundamental	339	25,4	391	35,7	332	24,3
Médio	719	51,7	468	48	721	49,8
Superior	326	22,9	355	16,4	406	25,9
Natureza do município						
Capital	384	24,3	415	26,8	381	24,8
Interior	760	57,9	593	58,7	818	58,1
Outros municípios da região metropolitana	240	17,8	206	14,4	260	17,1
Região de residência						
Centro-Oeste	120	7,7	88	8,3	603	7,9
Nordeste	359	26,2	336	27,2	221	26
Norte	118	7,9	102	8,9	392	8,1
Sudeste	586	43,5	518	41,6	123	43,1
Sul	201	14,7	170	14	120	14,9
Total	1.384	100	1.214	100	1.459	100

A Tabela 2 apresenta o consumo de alimentos em 2019, 2020 e 2021. Nos três ciclos, os subgrupos de alimentos *in natura* e minimamente processados (e suas preparações culinárias) mais consumidos foram os cereais (arroz, macarrão, polenta, milho ou cuscuz) e as leguminosas (feijão, ervilha, lentilha, soja ou grão de bico). Entre alimentos ultraprocessados, destaca-se o consumo de margarina e molhos industrializados (maionese, ketchup ou outros molhos industrializados), de refrigerante e de embutidos (salsicha, linguiça, mortadela, presunto ou outro alimento embutido). Comparando o consumo de alimentos nos três ciclos da pesquisa, observa-se, entre 2019 e 2020, aumento significativo no consumo de cereais, leite, salgadinhos de pacote ou biscoitos/bolachas salgadas e margarina e molhos industrializados, em contraponto à diminuição de consumo de ovos. Entre 2019 e 2021 e entre 2020 e 2021, por outro lado, observou-se diminuição significativa no consumo de cereais, hortaliças, frutas e sucos de fruta industrializados (suco de fruta em caixa, caixinha ou lata ou refresco em pó) e aumento no consumo de refrigerante, bolacha doce, recheada ou bolinho de pacote, embutidos, margarina e molhos industrializados e refeições prontas (macarrão instantâneo, sopa de pacote, lasanha congelada ou outro prato pronto comprado congelado).

**Tabela 2:** Prevalência do consumo de alimentos no dia anterior à pesquisa. Brasil, 2019, 2020 e 2021.

Grupos de alimentos	Ano da pesquisa					

	2019		2020		2021	
		
	%	EP	%	EP	%	EP
Alimentos *in natura* , minimamente processados e preparações culinárias						
Arroz, macarrão, polenta, milho ou cuscuz	83,8	1	91,3^a^	0,9	87,7^a,b^	0,9
Batata, mandioca/aipim/macaxeira, cará ou inhame	42,4	1,3	39,1	1,6	43	1,3
Feijão, ervilha, lentilha, soja ou grão de bico	75,8	1,2	75,9	1,4	74,5	1,2
Porco, carne, frango ou peixe	86	0,9	86,5	1,1	86,7	0,9
Ovo frito, cozido ou mexido	45,4	1,3	41,2^a^	1,6	44,1	1,3
Hortaliças	80,6	1,1	80,7	1,3	75,8^a,b^	1,2
Frutas	67,8	1,3	65	1,6	61,3^a^	1,3
Leite	54,2	1,4	59,0^a^	1,6	56	1,3
Alimentos ultraprocessados						
Refrigerante	33,2	1,3	32,7	1,5	42,4^a,b^	1,3
Suco de fruta em caixa, caixinha ou lata ou refresco em pó	29,7	1,2	29,3	1,5	26,3^a^	1,2
Bebida achocolatada ou iogurte com sabor	21,7	1,1	22,1	1,3	21,9	1,1
Salgadinho de pacote ou biscoito/bolacha salgada	30,1	1,2	35,0^a^	1,6	32	1,2
Biscoito/bolacha doce, recheada ou bolinho de pacote	33	1,3	31,3	1,5	36,7^a,b^	1,3
Sorvete, chocolate, gelatina, flan ou outra sobremesa industrializada	24,4	1,2	21,8	1,3	22,1	1,1
Salsicha, linguiça, mortadela, presunto ou outro alimento embutido	31,1	1,3	34,4	1,5	37,5^a^	1,3
Pão de forma, de cachorro-quente ou de hambúrguer	29,3	1,2	29,1	1,5	32,1	1,2
Margarina, maionese, ketchup ou outros molhos industrializados	50,2	1,4	54,4^a^	1,6	58,6^a,b^	1,3
Macarrão instantâneo, sopa de pacote, lasanha congelada ou outro prato pronto comprado congelado	9,7	0,8	10,9	1	13,9^a,b^	0,9

Fonte: dados do inquérito Datafolha, realizado com amostras representativas da população adulta brasileira. Para mais informações, vide métodos.

EP: erro-padrão.

^a^p = 0,05 (ano de referência: 2019).

^b^p = 0,05 (ano de referência: 2020).

A Figura 1 apresenta a distribuição de indivíduos de acordo com as principais mudanças na compra e no preparo de alimentos durante a pandemia de covid-19. Quando questionados, 46,3% dos entrevistados relataram que consumiam mais alimentos preparados em casa, 43,0% disseram que não houve alteração nos padrões de aquisição ou preparo dos alimentos e 9% relataram aumento em pedidos de refeições para entrega em casa.

**Figura 1:** Percepção de mudanças na compra e no preparo de alimentos durante a pandemia de covid-19. Brasil, 2021.

A Tabela 3 apresenta as alterações na alimentação durante a pandemia de covid-19 entre os indivíduos que referiram “consumir mais comida feita em casa” e “pedir mais refeições para entrega em casa”. Entre aqueles que consumiram mais refeições feitas em casa durante a pandemia, destaca-se a proporção de participantes que declararam aumento no consumo de ovo, frango, frutas, verduras e legumes e indivíduos que relataram diminuição do consumo de carne bovina. Adicionalmente, uma maior proporção de indivíduos relatou a diminuição no consumo de todos os subgrupos de alimentos ultraprocessados, em comparação à proporção de participantes que relatou aumento no consumo desses alimentos. Entre aqueles que informaram que pediram mais refeições para entrega em casa durante a pandemia, destaca-se a proporção de participantes que declarou aumento no consumo de carne de porco, ovo, refrigerante e refeições prontas congeladas e de indivíduos que relataram uma diminuição no consumo de carne bovina.

**Tabela 3:** Percepção de mudanças no consumo de alimentos (aumento, redução ou inalterações) durante a pandemia de covid-19 entre os indivíduos que elegeram, como principal mudança na compra e no preparo de alimentos, “consumir mais comida feita em casa” e “pedir mais refeições para entrega em casa”. Brasil, 2021.

Variáveis	Indivíduos que relataram consumir mais comida feita em casa durante a pandemia de covid-19 (n = 674)			Indivíduos que relataram pedir mais refeições para entrega em casa durante a pandemia de covid-19 (n = 133)		
	
	Aumentou	Reduziu	Não alterou	Aumentou	Reduziu	Não alterou
					
	(%)	(%)	(%)	(%)	(%)	(%)
Alimentos *in natura* , minimamente processados e preparações culinárias						
Carne de boi	22,3	47,8	29,8	20,1	40,8	39,1
Carne de porco	19,8	34,6	45,6	20,2	33,4	46,4
Frango	53,8	13,4	32,9	53,2	13	33,8
Peixe	26,2	31,3	42,6	21,8	26,6	51,6
Frutas	49,2	19,8	31,0	37,4	21,2	41,4
Verduras e legumes	47,2	19,0	33,9	30,5	22,5	46,9
Macarrão	29,1	28,6	42,3	34,0	18,8	47,3
Arroz	37,7	15,4	46,9	31,4	16,8	51,7
Feijão	37,9	14,9	47,2	29,6	19,8	50,6
Leite	29,2	29,1	41,6	29,3	20,1	50,7
Ovo	54,4	11,3	34,3	49,9	12,3	37,8
Alimentos ultraprocessados						
Refrigerante	17,5	47,5	35	44	24,6	31,3
Suco de fruta em caixa, caixinha ou lata ou refresco em pó	16,4	39,1	44,5	29,5	22,3	48,2
Bebida achocolatada ou iogurte com sabor	10,2	43,3	46,6	27,2	20,7	52,2
Salgadinho de pacote ou biscoito/bolacha salgada	15	44,7	40,3	29,3	27,3	43,4
Biscoito/bolacha doce, recheada ou bolinho de pacote	16,3	41,3	42,4	28,3	28	43,7
Sorvete, chocolate, gelatina, flan ou outra sobremesa industrializada	11,9	46,6	41,5	28,3	22,7	49
Salsicha, linguiça, mortadela, presunto ou outro alimento embutido	21,4	38,4	40,1	35,2	22,7	42,1
Pão de forma, de cachorro-quente ou de hambúrguer	20,1	40,2	39,7	39,1	20	40,8
Margarina, maionese, ketchup ou outros molhos industrializados	16	37	47	36,4	18,7	44,9
Refeições prontas congeladas (pizza, lasanha etc.)	8,6	46,2	45,2	44,4	21,7	33,9
Macarrão ou sopa instantâneos	19,9	34,1	46	28,7	28,9	42,4

Fonte: dados do inquérito Datafolha realizado com amostras representativas da população adulta brasileira. Para mais informações, vide métodos.

A Figura 2 apresenta a distribuição de indivíduos de acordo com a percepção de mudanças nos hábitos alimentares cujos principais motivos para alterações aconteceram durante a pandemia de covid-19. Quando questionados, 51,4% dos entrevistados relataram não ter alterado a alimentação durante a pandemia. Ademais, entre aqueles que relataram mudanças (48,6%), o motivo mais frequente foi a maior preocupação com a saúde (39,1%), seguido da diminuição da renda familiar (30,2%) e de mais tempo disponível para cozinhar (16,4%).

**Figura 2:** Percepção de mudanças nos hábitos alimentares e os principais motivos para essas alterações terem acontecido durante pandemia de covid-19, Brasil, 2021.

A Tabela 4 apresenta as percepções de mudanças no consumo de grupos e subgrupos de alimentos durante a pandemia de covid-19 entre os indivíduos que elegeram como principal razão para tais mudanças “maior preocupação com saúde”, “diminuição do orçamento familiar” e “mais tempo disponível para cozinhar em casa”. Entre aqueles que declararam se preocupar mais com a saúde, destaca-se um aumento no consumo de frango, frutas, verduras e legumes e ovo e uma diminuição no consumo de carne bovina e de todos os subgrupos de alimentos ultraprocessados. Entre aqueles que declararam diminuição do orçamento familiar, destaca-se aumento no consumo de frango e ovo e diminuição no consumo de carne bovina, carne de porco, peixe, frutas, leite e de todos os subgrupos de alimentos ultraprocessados. Entre aqueles que declararam mais tempo disponível para cozinhar em casa, destaca-se um aumento no consumo de frango, frutas, verduras e legumes, arroz, feijão e ovo. Adicionalmente, entre esses indivíduos, a maioria relatou que não houve alteração no consumo de grupos de alimentos ultraprocessados.

**Tabela 4:** Percepção de mudanças no consumo de alimentos (aumento, redução ou inalterações) durante a pandemia de covid-19 entre os indivíduos que elegeram, como principal razão para tais mudanças, “maior preocupação com saúde”, “diminuição do orçamento familiar” e “mais tempo disponível para cozinhar em casa”. Brasil, 2021.

Variáveis	Indivíduos que relataram maior preocupação com a saúde como a principal causa da mudança alimentar durante a pandemia de covid-19 (n = 288)			Indivíduos que relataram diminuição do orçamento familiar como a principal causa da mudança alimentar durante a pandemia de covid-19 (n = 207)			Indivíduos que relataram ter mais tempo para cozinhar em casa como a principal causa da mudança alimentar durante a pandemia de covid-19 (n = 123)		
		
	Aumentou	Reduziu	Não alterou	Aumentou	Reduziu	Não alterou	Aumentou	Reduziu	Não alterou
								
	(%)	(%)	(%)	(%)	(%)	(%)	(%)	(%)	(%)
Alimentos *in natura* , minimamente processados e preparações culinárias									
Carne de boi	23,4	48,7	27,8	7,1	76	16,8	33,5	28,1	38,4
Carne de porco	16,6	38,6	44,8	17,9	50,5	31,6	23,7	22,7	53,6
Frango	58,7	12,7	28,6	49,2	27,5	23,3	62,6	6,4	31
Peixe	32,4	25,8	41,9	14,1	49,3	36,6	34,8	24	41,3
Frutas	63,6	13,6	22,8	31,8	42	26,2	64	10,5	25,5
Verduras e legumes	63,6	12,8	23,6	34,5	34	31,4	68,1	10,6	21,2
Macarrão	27,4	33,9	38,7	30,8	32,2	37	26,9	23,1	50
Arroz	33,1	27,4	39,5	33,4	20,9	45,7	49,3	10,9	39,8
Feijão	39,3	17	43,7	31,3	26,9	41,8	44,3	8,5	47,3
Leite	30,7	26,2	43,1	25,1	40,3	34,6	37,2	17,3	45,5
Ovo	53,5	15	31,4	59,2	13,3	27,5	63,5	7,2	29,3
Alimentos ultraprocessados									
Refrigerante	19,4	50,7	29,9	16,2	58,2	25,6	25,6	33,2	41,2
Suco de fruta em caixa, caixinha ou lata ou refresco em pó	14,9	44	41,2	18,5	47,9	33,6	23,7	26,8	49,5
Bebida achocolatada ou iogurte com sabor	11,7	43,6	44,8	7,9	55,8	36,4	20,3	31	48,7
Salgadinho de pacote ou biscoito/bolacha salgada	11,4	52,9	35,7	19,4	54,7	25,9	25,2	36,1	38,7
Biscoito/bolacha doce, recheada ou bolinho de pacote	16,5	45,1	38,4	18,6	55,5	25,8	25,1	32,3	42,6
Sorvete, chocolate, gelatina, flan ou outra sobremesa industrializada	11,2	47,9	40,9	7,8	61	31,1	31,5	28,7	39,8
Salsicha, linguiça, mortadela, presunto ou outro alimento embutido	17,3	44	38,7	28,7	43,9	27,4	28,5	24,5	47
Pão de forma, de cachorro-quente ou de hambúrguer	21,4	39,2	39,5	19,1	48,7	32,2	27,9	24,4	47,7
Margarina, maionese, ketchup ou outros molhos industrializados	16,9	43,2	39,9	15,8	44,6	39,5	26,3	28,1	45,6
Refeições prontas congeladas (pizza, lasanha etc.)	7,7	47,1	45,2	10,1	60,1	29,9	23,8	35,1	41,1
Macarrão ou sopa instantâneos	16,8	42,6	40,6	21,2	39,2	39,6	14,6	30,9	54,5

Fonte: dados do inquérito Datafolha realizado com amostras representativas da população adulta brasileira. Para mais informações, vide métodos.

## DISCUSSÃO

Os resultados deste estudo sugerem mudanças negativas na alimentação da população brasileira durante a pandemia de covid-19. No entanto, observaram-se alterações diferentes em distintos estágios da pandemia.

No primeiro ciclo da pesquisa, realizado antes da pandemia (em 2019), mais de 75% dos participantes relataram consumo, no dia anterior à entrevista, de pelo menos um tipo de carne, cereal, hortaliça ou leguminosa. Grupos de alimentos ultraprocessados, embora relatados por uma parcela importante da população, apareceram em menor proporção de consumo. Esses resultados vão ao encontro de pesquisas nacionais que indicam que, apesar de um aumento crescente no consumo de alimentos ultraprocessados, a base da alimentação brasileira ainda é composta por alimentos *in natura* e minimamente processados, destacando o consumo de arroz, feijão e carne^13^.

O segundo ciclo da pesquisa indicou que, durante a pandemia, houve alterações no consumo de alimentos pelos brasileiros. Em 2020, cerca de três meses após o início do período de confinamento, observou-se um aumento na proporção de indivíduos que relataram consumo de cereais e leite no dia anterior à pesquisa. Também foi observado aumento no consumo de salgadinho de pacote ou biscoito/bolacha salgada e margarina e molhos industrializados, indicando aumento no consumo tanto de marcadores saudáveis quanto de não saudáveis da dieta.

Tais resultados são comparáveis a outras pesquisas conduzidas no país nos primeiros meses após a implementação de medidas restritivas. Entre participantes da coorte NutriNet Brasil, observou-se, de maneira geral, um aumento no consumo de marcadores saudáveis da alimentação, porém, entre estratos socioeconomicamente mais baixos ocorreu a tendência oposta, uma vez que foi relatado aumento no consumo de marcadores não saudáveis da dieta^3^. Outrossim, estudo observacional realizado em uma amostra de conveniência relatou aumento do consumo de *fast food* e refeições prontas em contraponto à diminuição do consumo de frutas^4^. Comparações com a literatura, entretanto, são limitadas pois os estudos utilizaram diferentes métodos para seleção de amostra e coleta de dados.

A comparação entre os primeiros ciclos (2019 e 2020) e o terceiro ciclo da pesquisa (2021) aponta um aumento do consumo de alimentos ultraprocessados, com destaque para refrigerantes, bolachas, embutidos, margarina e molhos industrializados e refeições prontas, em contraponto à diminuição do consumo de alimentos *in natura* e minimamente processados e suas preparações culinárias, especialmente de frutas e hortaliças. Devido ao curto período entre as pesquisas, as mudanças foram pequenas, porém estatisticamente significativas. Tais resultados preocupam, à medida que o consumo de alimentos ultraprocessados tem sido associado a um declínio no perfil nutricional da dieta^14^. Adicionalmente, estudos apontam associação entre o consumo de alimentos ultraprocessados e desfechos negativos em saúde, tal como obesidade, síndrome metabólica, diabetes e diferentes tipos de câncer^15^. A obesidade e outras doenças crônicas não transmissíveis (DCNT), por sua vez, são fatores de risco para o agravamento de sintomas em pessoas infectadas pela covid-19^19 , 20^.

Mudanças na alimentação durante a pandemia de covid-19 podem ser atribuídas a diferentes razões. Como observado nos resultados sobre percepções de mudanças na alimentação durante o confinamento, a maioria dos indivíduos que relatou “maior preocupação com saúde” afirmou ter aumentado o consumo de frutas e hortaliças e diminuído o consumo de alimentos ultraprocessados nesse período. Mudanças semelhantes foram encontradas entre indivíduos que declararam “consumir mais alimentos preparados em casa” como principal mudança na compra e no preparo de alimentos durante a pandemia. Indivíduos que declararam “pedir mais refeições para entrega em casa”, por outro lado, afirmaram, em sua maioria, ter aumentado o consumo de alimentos ultraprocessados, tais como refrigerante e refeições prontas congeladas.

Outros fatores não avaliados neste estudo também podem explicar o aumento do consumo de alimentos ultraprocessados. A publicidade desses produtos, por exemplo, é um dos fatores que pode ter impulsionado seu consumo durante a pandemia de covid-19. Estudos apontam que a indústria de alimentos ultraprocessados tirou proveito do contexto global para promover seus produtos como “vantajosos” durante a pandemia, uma vez que são menos perecíveis e, assim, poderiam contribuir com o distanciamento social, apelando para a responsabilidade social dos consumidores e vinculando sua imagem à saúde^21 , 22^.

O aumento do consumo de alimentos ultraprocessados também pode estar vinculado ao impacto negativo do distanciamento social na saúde mental da população. Estudos conduzidos no Brasil apontam alta ocorrência de sintomas psicológicos, como depressão, ansiedade e estresse, durante a pandemia^23 , 24^. Perturbações na saúde mental, por sua vez, têm sido associadas a dietas não saudáveis^25^, de modo que sintomas psicológicos podem, em parte, explicar o aumento do consumo desses alimentos durante o período de distanciamento social.

Outros fatores, como aumento do preço dos alimentos^29^, aumento da taxa da população desempregada^30^e redução do auxílio emergencial^10^durante a pandemia também podem explicar as mudanças no consumo alimentar. Neste estudo, indivíduos que relataram “diminuição do orçamento familiar” como principal causa da mudança alimentar durante a pandemia de covid-19 informaram, em sua maioria, a redução no consumo de alimentos *in natura* e minimamente processados como carne bovina e suína, peixe, frutas e leite, além do consumo de todos os grupos de alimentos ultraprocessados. Esse resultado sugere dificuldade de acesso a alimentos por uma parcela importante da população. Tais dados vão ao encontro aos resultados do “Inquérito nacional sobre insegurança alimentar no contexto da pandemia da covid-19 no Brasil”, que mostram que a pandemia acelerou a prevalência de insegurança alimentar no país, que apresentava projeções de crescimento desde 2013. No Brasil, entre 2018 e 2020, a insegurança alimentar leve aumentou de 20,7% para 34,7%; a insegurança alimentar moderada aumentou de 10,1% para 11,5%; e a insegurança alimentar grave aumentou de 5,8% para 9%^10^.

Dentre as limitações do presente estudo, destaca-se a aferição do consumo alimentar da população que foi realizada por meio de questionários simplificados em períodos específicos, que desconsideraram a sazonalidade anual de produção de alimentos. Adicionalmente, devido a restrições de circulação decorrentes da pandemia, o método de coleta das informações foi diferente no segundo ciclo da pesquisa, embora os questionários de consumo alimentar tenham sido os mesmos nos três ciclos. Também vale ressaltar que os resultados foram, parcialmente, baseados em percepções de mudanças no consumo alimentar, que não necessariamente refletem as alterações efetivas. Dentre os pontos positivos do estudo, destaca-se sua amostra representativa da população brasileira. Além disso, este é o primeiro estudo publicado a investigar mudanças na alimentação em dois pontos distintos da pandemia de covid-19 e a investigar as razões para tais mudanças.

Resultados deste estudo ajudarão a entender os efeitos da pandemia de covid-19 na alimentação da população e a compreender os motivos que levaram a tais alterações. O aumento do consumo de alimentos ultraprocessados já é um fenômeno reportado no Brasil por estudos nacionais realizados periodicamente^13^, entretanto, este estudo, assim como outros^3 , 4^, sugere que a pandemia acelerou esse fenômeno, impactando negativamente a dieta da população. Porém, mais pesquisas são necessárias para avaliar a magnitude e os efeitos da pandemia de covid-19 na alimentação dos brasileiros a longo prazo.
